# Ensemble Merit Merge Feature Selection for Enhanced Multinomial Classification in Alzheimer's Dementia

**DOI:** 10.1155/2015/676129

**Published:** 2015-10-20

**Authors:** T. R. Sivapriya, A. R. Nadira Banu Kamal, P. Ranjit Jeba Thangaiah

**Affiliations:** ^1^Department of Computer Science, Lady Doak College, Madurai, Tamil Nadu 625002, India; ^2^The Alzheimer's Disease Neuroimaging Initiative, San Diego, CA 92093-0949, USA; ^3^Department of Computer Science, TBAK College, Kilakarai, Tamil Nadu 623517, India; ^4^Department of Computer Applications, Karunya University, Coimbatore, Tamil Nadu 641114, India

## Abstract

The objective of this study is to develop an ensemble classifier with Merit Merge feature selection that will enhance efficiency of classification in a multivariate multiclass medical data for effective disease diagnostics. The large volumes of features extracted from brain Magnetic Resonance Images and neuropsychological tests for diagnosis lead to more complexity in classification procedures. A higher level of objectivity than what readers have is needed to produce reliable dementia diagnostic techniques. Ensemble approach which is trained with features selected from multiple biomarkers facilitated accurate classification when compared with conventional classification techniques. Ensemble approach for feature selection is experimented with classifiers like Naïve Bayes, Random forest, Support Vector Machine, and C4.5. Feature search is done with Particle Swarm Optimisation to retrieve the subset of features for further selection with the ensemble classifier. Features selected by the proposed C4.5 ensemble classifier with Particle Swarm Optimisation search, coupled with Merit Merge technique (CPEMM), outperformed bagging feature selection of SVM, NB, and Random forest classifiers. The proposed CPEMM feature selection found the best subset of features that efficiently discriminated normal individuals and patients affected with Mild Cognitive Impairment and Alzheimer's Dementia with 98.7% accuracy.

## 1. Introduction

Dementia is a neuropsychiatric disease widespread in many countries that affects people in older age [1]. Early diagnosis helps in palliative care, mitigation, and prevention of disease progression. Accurate diagnosis of crucial factors that cause the disease is vital for timely treatment [2]. Several high-dimensional pattern classification techniques have been built upon methods of computational anatomy, functional neuroimaging [3], and neuropsychological analysis demonstrating that classifications of individuals, in contrast to group analysis, can be achieved with relatively high classification accuracy. Recently there has been a growing interest for high-dimensional feature selection and classification methods that can combine information from the whole brain measurement [4] and neuropsychological data [5] to discriminate between individual subjects. Moreover another study indicates that not only older population but also men and women under the age of 50 are affected by dementia [6]. There are several studies that have proved the effective utilization of neuropsychological test data [7–9] for earlier diagnosis of dementia and for conversion from Mild Cognitive Impairment to Dementia.

The application of artificial intelligence techniques to cognitive measures provides enhanced feature specific analytic methods for neuropsychological data that has already been experimented for the diagnosis of dementia caused by Alzheimer's disease [10]. Automated classification of Dementia with PET images has been done with structural warping of neuroimaging data [11]. Klöppel et al. developed automated classification of Magnetic Resonance scans and compared the performance of computerized method with a radiologist in this area of research [12]. Larner has reviewed the importance of cognitive screening instruments and their accuracy in diagnosis of Dementia [13]. A diagnostic method was developed using neuropsychological test improved by multivariate analyses using PCA [7]. A research report comparing the conventional statistical classifiers and machine learning methods demonstrated the comparable improved performance of the machine learning methods [14]. A study by Quintana et al. provides evidence that Artificial Neural Networks can be a useful tool for the analysis of neuropsychological profiles related to clinical syndromes. Yu et al. developed a model of Support Vector Machine for prediction of common diseases in the case of occurrence of diabetes and prediabetes [15]. Hachesu et al. applied the Neural Networks, Decision Tree, and SVM to determine and predict the length of stay of cardiac patients [16].

Kabir et al. presented a new feature selection (FS) algorithm based on the wrapper approach using Neural Networks [17]. The vital aspect of this algorithm is the automatic determination of Neural Network architectures during the feature selection process. Maldonado et al. have applied SVM for simultaneous feature selection and classification [18]. New approach for classification of microarray high-dimensional data has been evolved [19]. Chen et al. applied classification trees for larger datasets in Bioinformatics [20]. Calle et al. developed a new strategy for genome data profiling with Random forest [21].

Several studies with multimodal data [22] have proven the classification efficiency of Random forest [14, 20, 21]. In a study for Differentiation of MCI from AD, Naïve Bayes, SVM, NN, and Decision Tree (DT) were used for feature selection and Naïve Bayes was used as the base classifier [23]. In that study, Naïve Bayes and DT gave better results when compared with SVM.

*Relevance of This Study.* Attribute selection performs a key role in building a good classifier which can efficiently delineate the patient records with absolute accuracy and efficiency. This study proposes an ensemble feature selection approach using J48 classifier with PSO search strategy and Merit Merge technique to do the following.

- Find the optimal subset that can effectively delineate the three classes as Normal (NL), Mild Cognitive Impairment (MCI), and Alzheimer's Dementia (AD) with ensemble feature selection.
- Find all possible subset combinations that can increase the accuracy in the discrimination of Mild Cognitive Impairment from Dementia.
- Train and test an ensemble model that can effectively classify multiclass medical data.

## 2. Feature Selection and Classification

### 2.1. Feature Selection

Feature selection is an important step that determines the performance of a classifier. Dimension reduction [24] is compulsory for better classification of larger datasets. Feature extraction selects the most relevant, nonredundant features of interest from the given data. In general, feature selection can be performed by filter, wrapper [17], and embedded methods. Several studies have been reported for feature selection with Support Vector Machine [18, 25, 26] and Random forest [21]. Uncu and Türksen developed a new approach with combination of filters and wrapper for feature selection [27].

Particle Swarm Optimisation (PSO) is a search technique that is a proven feature selection mechanism [28]. The capability of PSO is that it can search in a very large search space and find solutions quickly compared to other evolutionary search techniques like Genetic Algorithm. Optimisation of solution plays a great role in classification and clustering applications. PSO has been used not only for feature selection [29]; it has been applied for the optimization of parameters in machine learning algorithms like SVM.

### 2.2. Bagging

Bagging follows a bootstrap method of data selection for classification. It uses classifiers of the same type. Bagging follows sampling with replacement procedure for selecting a set of data as input for a classifier. Since it has classifiers of the same type, majority vote across the ensemble formulates the final result. Boosting ensemble follows a sequential method where every classifier is formed based on the output and error of the previously constructed classifier [30]. Second classifier performs better than the first and the same for the consecutively constructed classifiers. Hence it takes more time for model construction and complexity increases. Moreover it results in overfitting of the given data. Ensemble classifier is a supervised learning model [31] that employs the concept of a group of multiple classifiers to improve classification accuracy. It combines many weak learners in order to generate a strong learning algorithm. The aim of applying ensemble method is to overcome the risk of overfitting by individual classifier.

### 2.3. Classification

#### 2.3.1. Support Vector Machines

Support Vector Machines (SVMs) were introduced in 1995 by Cortes and Vapnik [32]. In terms of theory the SVMs are well founded and proved to be very efficient in classification tasks. The advantages of such classifiers are that they are independent of the dimensionality of the feature space and that the results obtained are very accurate, although the training time is very high. Support Vector Machines are feed-forward networks with a single layer of nonlinear units. Their design has good generalization performance as an objective and follows for that reason the principle of structural risk minimization that is rooted in VC dimension theory.

The training points, for which the equality of the separating plane is satisfied, that is,

(1) $$\begin{array}{c} \forall i,\;y_{i}\left(x_{i}\cdot w+b\right)\geq 0, \end{array}$$

those which wind up lying on one of the hyperplane *H*_1_, *H*_2_, and whose removal would change the solution found, are called Support Vectors (SVs). This algorithm is firmly grounded in the framework of statistical learning theory, Vapnik-Chervonenkis (VC) theory, which improves the generalization ability of learning machines to unseen data. In the last few years Support Vector Machines have shown excellent performance in many real-world applications including object recognition, face detection, and dementia diagnosis in images.

#### 2.3.2. Random Forest

Random forest trees introduced by Breiman [33] are a method of building a forest of uncorrelated trees with randomized node optimization and bagging. Out of bag errors is used as an estimate of the generalization error. Random forest (RF) is used to measure variable importance through permutation [34]. The general technique of bootstrap aggregation is applied in the training algorithm. In Random forest implementation only the number of trees in the forest and the number of attributes for prediction need to be defined [35].

#### 2.3.3. C4.5

C4.5 algorithm is used to generate a Decision Tree that can be used for classification problems [36]. Decision Tree is built using the entropy value obtained from the given data. C4.5 uses binary split or multivalued split in selection of attributes. Performance of the algorithm varies with cross validation and train-test method. The average accuracy across several folds should be taken as the evaluation measure. As with all other classifiers, precision and recall increases with more records in the training dataset. J48 is the Java implementation of C4.5 in Weka tool. C4.5 is an improvement of the ID3 algorithm and is capable of handling both discrete and continuous values. Another advantage is that fields with missing values need not be imputed with any values. Rather that field will not be used for calculation of entropy and information gain.

#### 2.3.4. Naïve Bayes

Naïve Bayes classifier is a statistical technique [37] that is applied for classification in data mining problems. It is based on probabilistic outcomes of a given data. It is a supervised learning technique and hence prior knowledge can be incorporated in its learning process. Hence it is well suited for medical diagnostics where the knowledge of the domain expert can be incorporated in prior in order to achieve higher performance.

## 3. Experimental Design

The reason for selection for C4.5 classifier is that it provides better accuracy when compared with Random forest, Naïve Bayes, and Support Vector Machine in multiclass classification problems. Ensemble feature selection is done with C4.5, SVM, RF, and NB followed by classification with C4.5. AdaBoost has the disadvantage of overfitting and the model construction involved more time and complexity. Hence bagging approach is selected for the multiclass dataset classification.

### 3.1. Dataset

Data used in the preparation of this paper were obtained from the Alzheimer's Disease Neuroimaging Initiative (ADNI) database (adni.loni.usc.edu). The ADNI was launched in 2003 as a public-private partnership, led by Principal Investigator Michael W. Weiner, MD. The primary goal of ADNI has been to test whether serial Magnetic Resonance Imaging (MRI), positron emission tomography (PET), other biological markers, and clinical and neuropsychological assessment can be combined to measure the progression of Mild Cognitive Impairment (MCI) and early Alzheimer's disease (AD).  Table 1 shows the details of data sets used in the study.  Table 2 lists the attributes in the dataset.

### 3.2. Preprocessing

Preprocessing precedes classification for noise removal and missing data management. Data was partitioned based on the month of visit. Records in each partition are clustered based on the diagnosis in that visit. Data was normalized with *z*-score normalization. Values of selective attributes were normalized to a range from 0 to 1. In prediction of length of stay of patients, classwise mean values of respective classes were used to replace numeric missing values and mode of different classes replaced nominal or ordinal missing values. Moving average (MA) operators are used for handling missing values in time series data. MA has been applied for medical data and nonstationary signals also [38]. Expectation maximization (EM) algorithm was used to impute the missing data in a study [39]. EM has already been applied in the analysis of Alzheimer's data and found to be more effective than multiple imputation methods [40]. Attributes with more than 40% missing data were removed from the attribute set to avoid misclassification and bias.

### 3.3. Ensemble Feature Selection

There are 3 phases in the proposed Merit Merge feature selection technique. Base classifier to be applied for feature selection is determined in Phase I by comparing the classifiers reported in the literature with the ensemble classifiers. After the identification of base classifier, PSO search is coupled with ensemble classifiers to identify feature sets with higher merit. The ensemble model is trained and tested with feature set to obtain the optimal subset that can be used for the multinomial classification.

**Figure 1:**
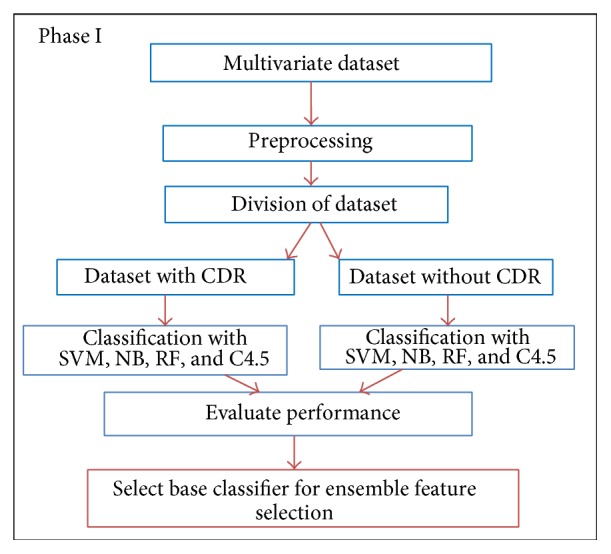
Selection of base classifier for ensemble feature selection.

*Phase I.* This phase determines the base classifier that can be used for modelling the ensemble classification model. Clinical dementia ratio is a key attribute in the discrimination of NL, MCI, and AD. Hence that key attribute is removed and the performance of classifiers is compared. It was noted in the previous study that classification by NB outperformed SVM. Hence those classifiers are compared with C4.5 in the classification of our multiclass problem.  Figure 1 shows the steps in the selection of base classifier. Data set containing both neuropsychological test data and neuroimaging measures with 870 instances was classified by NB, RF, SVM, and C4.5 decision tree. Data set without clinical dementia ratio attribute is again classified with the four classifiers. This is done to evaluate the sensitivity of the classifier even in the absence of relevant attributes. Since C4.5 decision tree classifier outperformed the other classifiers used in the multiclass classification, ensemble approach with PSO search is proposed and tested in this work. Naïve Bayes provided a better accuracy compared to RF and SVM. Ensemble feature selection is performed with C4.5 tree having binary split and pruning with minimum description length technique. Random forest ensemble is implemented with 100 to 1000 trees. Out of bag error reduced and remained constant with 600 and more number of trees. Support vector machine is implemented with LIBSVM. The Radial Basis Function (RBF) kernel was used for classification. RBF kernel showed higher accuracy than other kernels. Kernel parameters C and *γ* values are optimized with grid search.

Given pair of values *x*_*i*_, *x*_*j*_, RBF kernel to find the separating hyperplane is defined as follows:

(2) $$\begin{array}{c} k\left(\;x_{i},x_{j}\;\right)=\exp\left(\;-\gamma {\left\|\;x_{i}-x_{j}\;\right\|}^{2}\;\right). \end{array}$$

It was observed that the sensitivity of J48 for each class was higher, compared to NB, SVM, and RF. Hence J48 is selected as the base classifier for feature selection and classification. J48 is the base ensemble classifier used in CPEMM.

**Figure 2:**
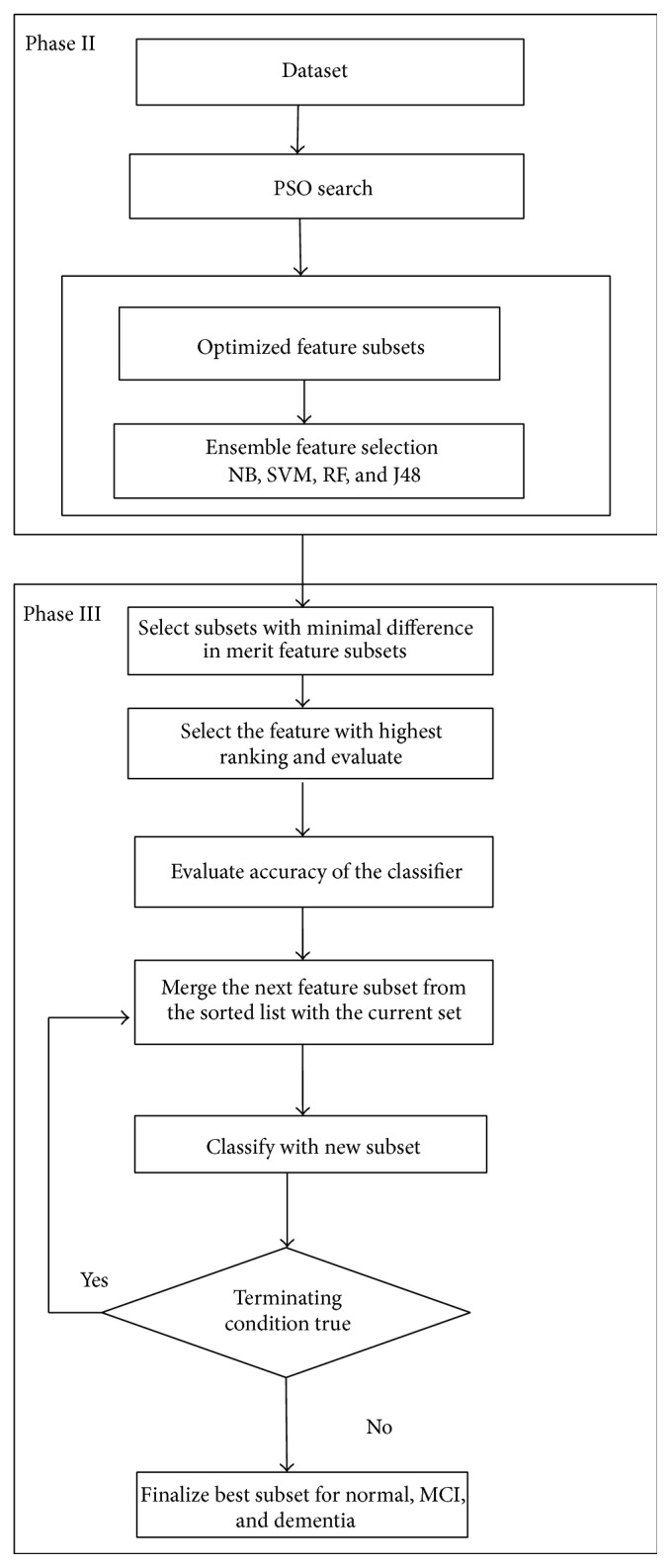
Steps in Phase II and Phase III.

*Phase II.* An overview of the steps in Phases II and III is presented in Figure 2. Ensemble feature selection is performed with C4.5 having binary split and pruning. Number of iterations in PSO search done is experimented in the range 60–100. Feature subsets were reduced in size as the number of iterations increased. With smaller number of iterations, ensemble search selected subsets with more features. As the iterations increased to find the best optimal solution, PSO resulted in generating subsets with lesser number of features. PSO search combined with NB, RF, and C4.5 ensembles generated the feature subsets. Feature subsets were sorted based on merit given by the search technique. It was observed that C4.5 ensemble selected the optimum subsets with Particle Swarm Optimisation with minimum number of iterations compared with NB and RF. RF ensemble returned good subsets in 2-class dataset. Binary split at node implemented in C4.5 selected relevant features with minimum iterations. CPEMM technique is presented as an Algorithm 1 following the overview of Phases II and III in Figure 2. 

*Phase III.* For each classifier, subset with the highest merit is considered for evaluation by the base classifier C4.5 and the accuracy is stored for further comparison.

**Case 1 .**

If subsets have equal merit, each subset is evaluated individually and also as a single subset after merging. If the merged subset does not increase accuracy, individual subsets are selected as relevant feature set.

**Case 2 .**

If more than 50% of subsets at the top of the sorted subset list have the same merit, the number of iterations is increased to get a minimal feature subset for evaluation. One limitation is that if the increase in iterations did not return reduced subset, this case should be probed further for enhancing feature selection.

**Case 3 .**

If there is a successive subset with much lower merit, the search for subset is terminated.

5-fold cross validation ensured that all the instances are used in the model development [41]. Alternate records are left out and trained with remaining records in every consecutive execution of the loop. Ensemble classifiers which are implemented in Weka tool is run in Pentium processor with 2.53 Ghz speed, 4 GB RAM, and 64 bit operating system. Statistical analysis of feature selection methods and performance of classifiers were implemented in *R*.

## 4. Results

The results are evaluated based on the performance of the classifier by feeding the different sets of feature set selected by

- C4.5, NB, and RF coupled with PSO,
- features selected by the CPEMM approach.

Accuracy, precision and recall of the classifier is evaluated with four datasets listed in Table 3.

### 4.1. Performance Measures

All classification results could have an error rate and will either fail to identify dementia or misclassify a normal patient as demented. It is common to describe this error rate by the terms True Positive and False Positive and True Negative and False Negative as follows.

True Positive (TP) is as follows: the result of classification is positive in the presence of the clinical abnormality. True Negative (TN) is as follows: the result of classification is negative in the absence of the clinical abnormality. False Positive (FP) is as follows: the result of classification is positive in the absence of the clinical abnormality. False Negative (FN) is as follows: the result of classification is negative in the presence of the clinical abnormality.

Accuracy defines the overall correctness of the model. Precision defines the number of correct classification obtained for each class. Its value falls between 0 and 1. Recall corresponds to the True Positive rate. Consider

(3) Accuracy=No. of correct classificationTotal no. of classification,

(4) Precision=TPTP+FP,

(5) Recall=TPTP+FN.

Receiver optimistic curves (ROC) are used to analyse the prediction capability of machine learning techniques used for classification and clustering [42]. ROC analysis is a graphical representation comparing the True Positive rate and False Positive rate in classification results. Area under the curve (AUC) characterizes the ROC of a classifier. The larger the value of AUC is, the more effective the performance the classifier will be. Press' *Q* test was used to evaluate the statistical significance of the difference in accuracy yielded by the classifiers. Given “*m*” samples, “*n*” correct classification, and “*p*” groups, test statistic was evaluated as follows:

(6) $$\begin{array}{c} Q=\frac{{\left(m-np\right)}^{2}}{m\left(p-1\right)}\sim \chi^{2}. \end{array}$$

**Figure 3:**
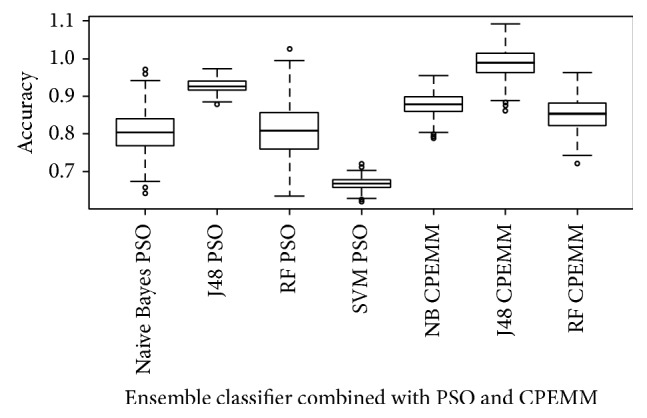
Accuracy of classifiers with features selected by PSO, CPEMM methods.

Naïve Bayes, C4.5 Decision Tree, Random forest, and SVM yielded statistically significant accuracy. It was found that feature selection by CPEMM considerably increased the percentage of records that were correctly classified. C4.5 classifier combined with CPEMM methodology provided the highest statistically significant difference in performance when compared with PSO and the conventional ensemble based feature selection technique as shown in Figure 3. Higher Median of 0.987 was yielded by the proposed combination of CPEMM and C4.5 ensemble classifier, while the other classifiers tested, that is, NB, RF, and SVM, had a median approximately above 0.75 to 0.88.

With C4.5 as base classifier, features selected by PSO in combination with NB, SVM, C4.5, and RF ensemble are listed in Table 3. CPEMM method is applied to merge subsets based on merit. The resultant subsets from each dataset are evaluated with C4.5 classifier. The accuracy obtained for each class (NL, AD, and MCI) is evaluated in each dataset. Sensitivity of classifier to the multiclass classification using the CPEMM approach is tabulated in Table 4.

CPEMM was applied to the feature sets obtained by NB, C4.5, and RF since the sensitivity of the SVM classifier was very low compared with other classifiers. The nonlinear RBF kernel was the best fitting kernel with SVM. Yet the accuracy obtained was below 70%. Hence the CPEMM strategy is applied and tested with NB, C4.5, and RF.

**Figure 4:**
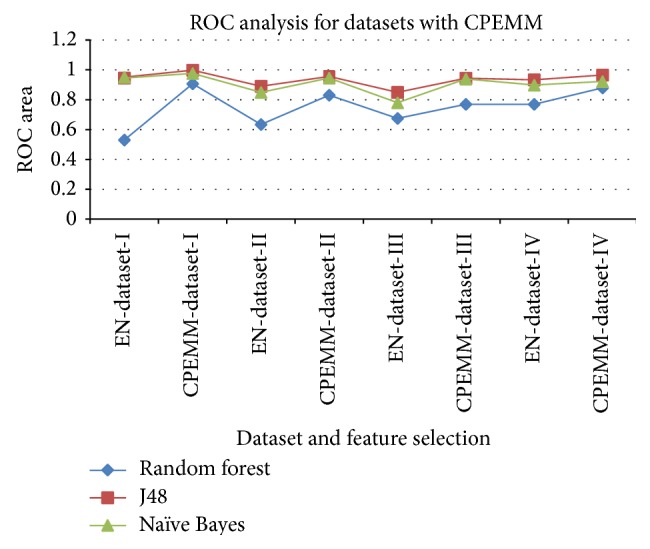
Comparison of area under the curve obtained by ordinary ensemble vs CPEMM for the four datasets.

The discriminating efficiency of J48 with respect to the three classes Normal, Dementia, and Mild Cognitive Impairment is evaluated. Classification of Normal class had higher sensitivity, compared to the delineation of Mild Cognitive Impairment and Dementia. The results are given in Table 4. Ensemble feature selection returned list of subsets with higher merit. CPEMM technique merged and evaluated the accuracy of successive subsets with higher merits. Efficiency of the classifier with features selected using CPEMM and the features selected with conventional ensemble feature selection is given as a comparison through ROC analysis in Figure 4. ROC area that is obtained with the four datasets is plotted in the graph. ROC of individual ensemble feature selection is plotted with ROC obtained with CPEMM.  Table 5 describes the features of the ensemble model for classification.

CPEMM yielded higher area under the curve values for all the four datasets experimented in our study.

## 5. Conclusion

C4.5 classifier provided better accuracy and sensitivity in multiclass classification of Alzheimer's Dementia. Ensemble of C4.5 classifier selected best fit subset for the evaluation of the three different classes with the highest Recall value 98.7 for the class MCI. It was evident that features selected by the C4.5 algorithm further increased the performance of Random forest and Naïve Bayes classifier also. The proposed ensemble with PSO search selected the minimal subset that is needed for the discrimination of diseases. Merit Merge approach further enhanced the feature selection by identifying the effective consolidated subset that could be used for the clinical diagnosis of dementia and Mild Cognitive Impairment that will lead to dementia. Our work also confirmed the fact that performance of SVM in the delineation of Mild Cognitive Impairment and Dementia is very low compared to Random forest, Naïve Bayes, and C4.5 algorithm as mentioned by Williams et al. [23]. Although the performance of Random forest was comparable to C4.5 and NB in the discrimination of 2-class data, accuracy of approximately 75% was provided for the 3-class problem. CPEMM was able to predict the relevant features for all datasets especially the CIDS. The proposed split and merge ensemble approach can be applied for any 3-class classification problem. It can be extended for the classification of high-dimensional datasets like microarray data also with preliminary feature reduction.

Classification with NB for discrimination of Dementia and MCI by previous study resulted in accuracy of around 80% and sensitivity of approximately 70% [14, 23]. Our CPEMM based on Bagging ensemble of J48 with Merit Merge technique yielded higher accuracy of 98.7% in train and test method [43]. Bagging approach with learning from more than one classifier found the minimal subset for effective diagnostics. Merit Merge approach found highly relevant, all possible subsets that contribute towards the multiclass classification. Proposed approach yielded a statistically significant difference with a mean area under the curve of approximately 0.977 in the multivariate classification of Dementia.

Bagging ensemble models provide a promising, error free, statistically significant machine learning method for disease diagnosis. The proposed methodology can be for applied disease state prediction even with class imbalanced datasets.

## Figures and Tables

**Algorithm 1:**
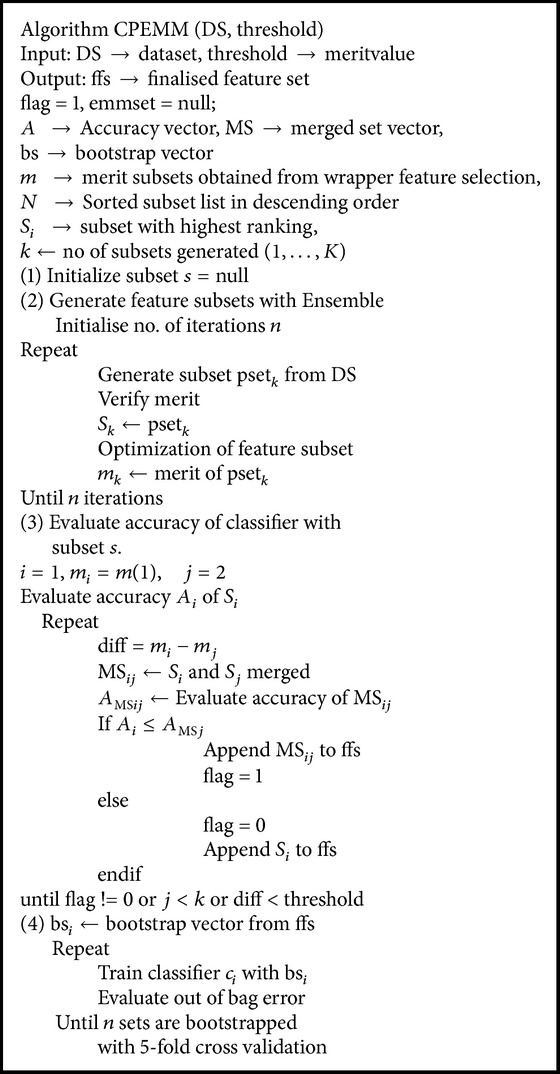


**Table 1:** Datasets used in the study.

Dataset	Number of instances	Number of attributes	Number of classes	AD	Normal	MCI
Neuropsychological dataset	750	48	3	150	200	400
Neuroimaging dataset	650	108	2	250	250	200
Baseline combined data	870	65	3	140	280	450
Combined dataset	750	40	2	150	200	400

**Table 2:** List of attributes derived from neuropsychological test and neuroimaging measures.

Neuropsychological and neuroimaging measures	
Average FDG-PET of angular, temporal, and posterior cingulate	Mini Mental State Examination-baseline

Average PIB SUVR of frontal cortex, anterior cingulate, precuneus cortex, and parietal cortex	Ventricles measure

Average AV45 SUVR of frontal, anterior cingulate, precuneus, and parietal cortex relative to the cerebellum	Hippocampus-baseline, volume

Clinical dementia ratio-SB	Whole brain-baseline, volume

ADAS 11	UCSF entorhinal-baseline, volume

ADAS 13	UCSF fusiform-baseline, volume

Mini Mental Scale Examination score	UCSF Med Temp-baseline

RAVLT (forgetting)	UCSF ICV-baseline

RAVLT (5 sum)	MOCA-baseline

Functional Assessment Questionnaire	Pt ECog-Memory-baseline

MOCA	Pt ECog-Language-baseline

Pt ECog-Memory	Pt ECog-Vis/Spat-baseline

Pt ECog-Language	Pt ECog-Plan-baseline

Pt ECog-Visual	Pt ECog-Organ-baseline

Pt ECog-Plan	Pt ECog-Div atten-baseline

Pt ECog-Organ	Pt ECog-Total-baseline

Pt ECog-Div atten	SP ECog-Mem-baseline

Pt ECog-Total	SP ECog-Lang-baseline

SP ECog-Memory	SP ECog-Vis/Spat-baseline

SP ECog-Language	SP ECog-Plan-baseline

SP ECog-Visual	SP ECog-Organ-baseline

SP ECog-Plan	SP ECog-Div atten-baseline

SP ECog-Organ	SP ECog-Total-baseline

SP ECog-Attention	Average FDG-PET of angular, temporal, and posterior cingulate at baseline

SP ECog-Total	Average PIB SUVR of frontal cortex, anterior cingulate, precuneus cortex, and parietal cortex at baseline

UCSF ventricles measures	Average AV45 (PET ligand) SUVR of frontal, anterior cingulate, precuneus, and parietal cortex relative to the cerebellum at baseline

UCSF hippocampus measure	CDR-SB

UCSF whole brain measure	ADAS 11, baseline

UCSF entorhinal measure	ADAS 13, baseline

UCSF fusiform measure	

UCSF temporal measure	RAVLT (forgetting), baseline

UCSF ICV	RAVLT (5 sum), baseline

Pt: patient, ECog: everyday cognition test, SP: study partner, ADAS: Alzheimer's disease assessment scale, MOCA: Montreal Cognitive Assessment, Ray Auditory Verbal Learning Test, ICV: intracranial volume, SUVR: Standard Uptake value ratio, and CDR-SB: Clinical Dementia Rating Sum of Boxes.

**Table 3:** Results for the 3-class dataset with ensemble of NB, J48, RF, SVM and with CPEMM ensemble.

	NB Ensemble			J48 Ensemble			RF Ensemble			SVM Ensemble			NB-CPEMM			J48-CPEMM			RF-CPEMM		
	Acc	Pre	Rec	Acc	Pre	Rec	Acc	Pre	Rec	Acc	Pre	Rec	Acc	Pre	Rec	Acc	Pre	Rec	Acc	Pre	Rec
NSDS	0.838	0.854	0.838	0.911	0.895	0.889	0.936	0.941	0.799	0.647	0.564	0.677	0.899	0.904	0.912	0.932	0.958	0.933	0.936	0.941	0.799
NIDS	0.756	0.737	0.556	0.936	0.941	0.937	0.545	0.645	0.545	0.667	0.685	0.734	0.834	0.895	0.738	0.904	0.934	0.907	0.545	0.645	0.545
CIDS-I	0.863	0.833	0.823	0.916	0.933	0.923	0.963	0.963	0.963	0.673	0.699	0.788	0.896	0.812	0.822	0.987	0.955	0.963	0.963	0.963	0.963
CIDS-II	0.756	0.654	0.563	0.945	0.931	0.924	0.797	0.723	0.634	0.685	0.676	0.657	0.885	0.823	0.813	0.971	0.965	0.923	0.888	0.856	0.833

(NSDS: Neuropsychological data set, NIDS: Neuroimaging dataseta, CIDS: combined dataset).

Acc: Accuracy, Pre: precision, Rec: Recall.

**Table 4:** Results with the maximal feature subset obtained by divide and merge feature selection technique.

Classifier	Normal class			Dementia			Mild Cognitive Impairment		
	Acc	Pre	Rec	Acc	Pre	Rec	Acc	Pre	Rec
J48	0.963	0.963	0.963	**0.978**	**0.953**	**0.967**	0.966	0.968	0.987
J48-TT	0.983	0.972	0.977	**0.986**	**0.973**	**0.963**	0.977	0.976	0.977
J48-CV	0.965	0.954	0.945	**0.966**	**0.956**	**0.955**	0.964	0.954	0.945

TT: training and testing; CV: cross validation.

**Table 5:** Description of the J48 ensemble model used for the multiclass classification.

Details	Value
Split method	Binary split

Cross validation accuracy	0.976

AUC with CV	0.971

Train and test accuracy	0.986

AUC with train and test	0.987

Common features selected by all methods	MMSE, CDR, hippocampus volume, and everyday cognition measures

Features added by CPEMM	Entorhinal measures, CDRSB, and Ray Auditory Verbal Learning Test-immediate
